# Improved PCR Amplification of Broad Spectrum GC DNA Templates

**DOI:** 10.1371/journal.pone.0156478

**Published:** 2016-06-07

**Authors:** Nicholas Guido, Elena Starostina, Devin Leake, Ishtiaq Saaem

**Affiliations:** Research & Development, Gen9 Inc, Cambridge, Massachusetts, United States of America; Naval Research Laboratory, UNITED STATES

## Abstract

Many applications in molecular biology can benefit from improved PCR amplification of DNA segments containing a wide range of GC content. Conventional PCR amplification of DNA sequences with regions of GC less than 30%, or higher than 70%, is complex due to secondary structures that block the DNA polymerase as well as mispriming and mis-annealing of the DNA. This complexity will often generate incomplete or nonspecific products that hamper downstream applications. In this study, we address multiplexed PCR amplification of DNA segments containing a wide range of GC content. In order to mitigate amplification complications due to high or low GC regions, we tested a combination of different PCR cycling conditions and chemical additives. To assess the fate of specific oligonucleotide (oligo) species with varying GC content in a multiplexed PCR, we developed a novel method of sequence analysis. Here we show that subcycling during the amplification process significantly improved amplification of short template pools (~200 bp), particularly when the template contained a low percent of GC. Furthermore, the combination of subcycling and 7-deaza-dGTP achieved efficient amplification of short templates ranging from 10–90% GC composition. Moreover, we found that 7-deaza-dGTP improved the amplification of longer products (~1000 bp). These methods provide an updated approach for PCR amplification of DNA segments containing a broad range of GC content.

## Introduction

*De novo* gene synthesis relies on the chemical synthesis of oligonucleotides that are used as the building blocks for enzymatic assembly, making it possible to synthesize multi-kilobase genes and even whole genomes [1,2,3]. This technology is now more affordable than actually cloning genes and it enables the use of existing genome databases to construct any intended target. Such targets can include synthetic enzymes, reporter genes, regulatory elements, and even entire pathways [4,5,6,7]. However, most gene synthesis approaches utilize PCR amplification, which has shown to be difficult for DNA templates with extreme percent GC content (i.e: greater than 75%). These sequences can be problematic during PCR amplification as they form hairpins and various other secondary structures leading to premature termination of the polymerase extension yielding incomplete and non-specific products [8].

Previously, researchers changed cycling conditions to overcome low and high GC DNA sequences. Subcycling of the annealing and elongation steps during PCR amplification of long DNA segments was previously shown to achieve efficient amplification especially when the segments contain a variety of GC content [9]. For example, this method has been successfully used to detect an inversion mutation of intron 22 in the Factor VIII gene in patients with severe hemophilia A [10,11,12,13], a region of the gene known for containing long sequences with high and low GC content.

Additionally, researchers have also overcome amplification unevenness, or bias, in a multiplexed PCR due to extreme GC content by performing PCR reactions in presence of organic molecules like dimethyl sulfoxide (DMSO), Betaine, glycerol, or 7-deaza-dGTP. These additives improve the amplification specificity and yield [14,15,16]. Recently, researchers have reported that a combination of either two (betaine and DMSO)[17] or three additives (betaine, DMSO, and 7-deaza-dGTP)[18] resolve the complex secondary structure formation found in GC-rich sequences and synergistically enhance their amplification. To date, no better method has been clearly defined for *de novo* synthesis of genes containing a broad range of GC contents.

In this study, we use a single-shot assembly process to synthesize genes. The process includes PCR amplification of pools of multiple short DNA templates differing in GC contents that often results in unsuccessful amplification. In order to achieve a uniform, or non-biased, amplification of the templates, we combined the subcycling protocol with chemical additives to expand the viable amplification range of short templates by greater than 10% on both ends of the GC spectrum. These pools of short templates were subsequently assembled into discrete longer templates (~1000 bp), where we further show that 7-deaza-dGTP particularly improved the amplification product specificity at the high GC end of the spectrum. This new method for PCR amplification of DNA segments with a broad range of GC sequences allows for efficient production of a variety of gene constructs.

## Materials and Methods

### Oligonucleotide pools

The oligonucleotide pools used in these experiments were synthetic DNA designed to have the desired GC content. Sequences are provided in supporting information S1 File. Synthesis was performed using Agilent’s Sureprint Oligonucleotide Library Synthesis (OLS) technology [19]. The work presented in this publication utilized a small portion of an OLS design. Oligonucleotides contained 10% GC to 90% GC with the bases distributed randomly throughout the molecules. The pools were mixed such that each pool would have the range of oligonucleotides with differing GC content to test many combinations in one multiplexed PCR. A single-shot assembly process was used to assemble synthesized oligonucleotides into larger genes [20].

### PCR conditions

The subcycling PCR of the short oligonucleotides was carried out with the Phusion HF polymerase (Thermo Fisher Scientific, Inc) as well as with the KAPA HotStart ReadyMix (Kapa biosystems). For the Phusion PCR without additives we used 200μM dNTPs and a 1x final concentration of the Phusion 5x HF buffer from Thermo Fisher Scientific, Inc. The KAPA PCR was carried out with the master mix in accordance to what is indicated in the KAPA literature. To address high GC content various additives were included in the Phusion HF PCR reactions as follows: 7-deaza-dGTP (NEB) at a 40:60 ratio with normal dGTP, as well as 50:50 and 60:40 ratios keeping the final concentration of dNTPs constant; DMSO (Sigma) at a final concentration of 2.5%, 5%, and 10%; betaine (Sigma) at a final concentration of 1M, 2M and 4M. Oligonucleotide pools were amplified using one common set of primers in the multiplexed amplification as to avoid bias from unique primers for each sequence. These primers (TGCAACCCCCAAGACAACGT, TGGTTGACTCTTGTGCCGCA) are 20 bases in length with a melting temperature of 65°C and GC content of 55%.

The PCR amplification reactions for the short oligonucleotides of varying GC content were based on the subcycling protocol previously mentioned [9] with the following thermocycle conditions: 95°C for 5 min, 29 cycles at 98°C for 20 sec, with 4 subcycles of 60°C for 15 sec and 65°C for 15 sec, a final extension of 65°C for 5 min and held at 12°C. The standard PCR protocol for the short oligonucleoties to which we compared our subcycling conditions was: 98°C for 30 sec, 40 cycles of 98°C for 5 sec, 65°C for 10 sec and 72°C for 10 sec, then 72°C for 3 min. No additives were used in these PCR reactions.

The longer fragments were amplified with KOD polymerase master mix (EMD Millipore). Conditions for amplification of longer fragments were: 98°C for 30 sec, 40 cycles of 98°C for 5 sec, 65°C for 10 sec and 72°C for 10 sec, then 72°C for 3 min.

### MiSeq sequencing and analysis

In order to be able to determine the relative representation of each individual oligonucleotide in a multiplexed PCR we used the Illumina MiSeq sequencer to identify individual sequences and effectively count the number of appearances of each unique sequence. These oligonucleotides were first labeled with Illumina adapters such that the primers for labeling had a region identical to the oligonucleotide primers listed above, and to the adapters appended to the ends (TCGTCGGCAGCGTCAGATGTGTATAAGAGACAGTGCAACCCCCAAGACAACGT, TCGTCGGCAGCGTCAGATGTGTATAAGAGACAGTGCAACCCCCAAGACAACGT). The labeling PCR was carried out using NEB Phusion master mix with the following thermocycle: 95°C for 2 min, 5 cycles of 95°C for 30 sec, 60°C for 30 sec and 72°C for 2 min, and finally 72°C for 5 min. The labeled material was SPRI cleaned using 1.8x Agencourt AMPure XP beads (Beckman Coulter Inc.) and placed into an indexing reaction as indicated in the Illumina MiSeq prep instructions. Due to the length of these oligonucleotides they were loaded onto the MiSeq at 3pM. The data were analyzed using in house software.

### GX Analysis

Capillary electrophoresis DNA analysis post amplification was done with the LabChip GX instrument from Perkin Elmer. The DNA 5K/RNA/CZE LabChip was used in the GX instrument as appropriate for the sizes of DNA being analyzed. LabChip GX software version 4.1 was used to analyze the GX data.

## Results

### Subcycling improves amplification of DNA templates containing low GC content

**Fig 1:**
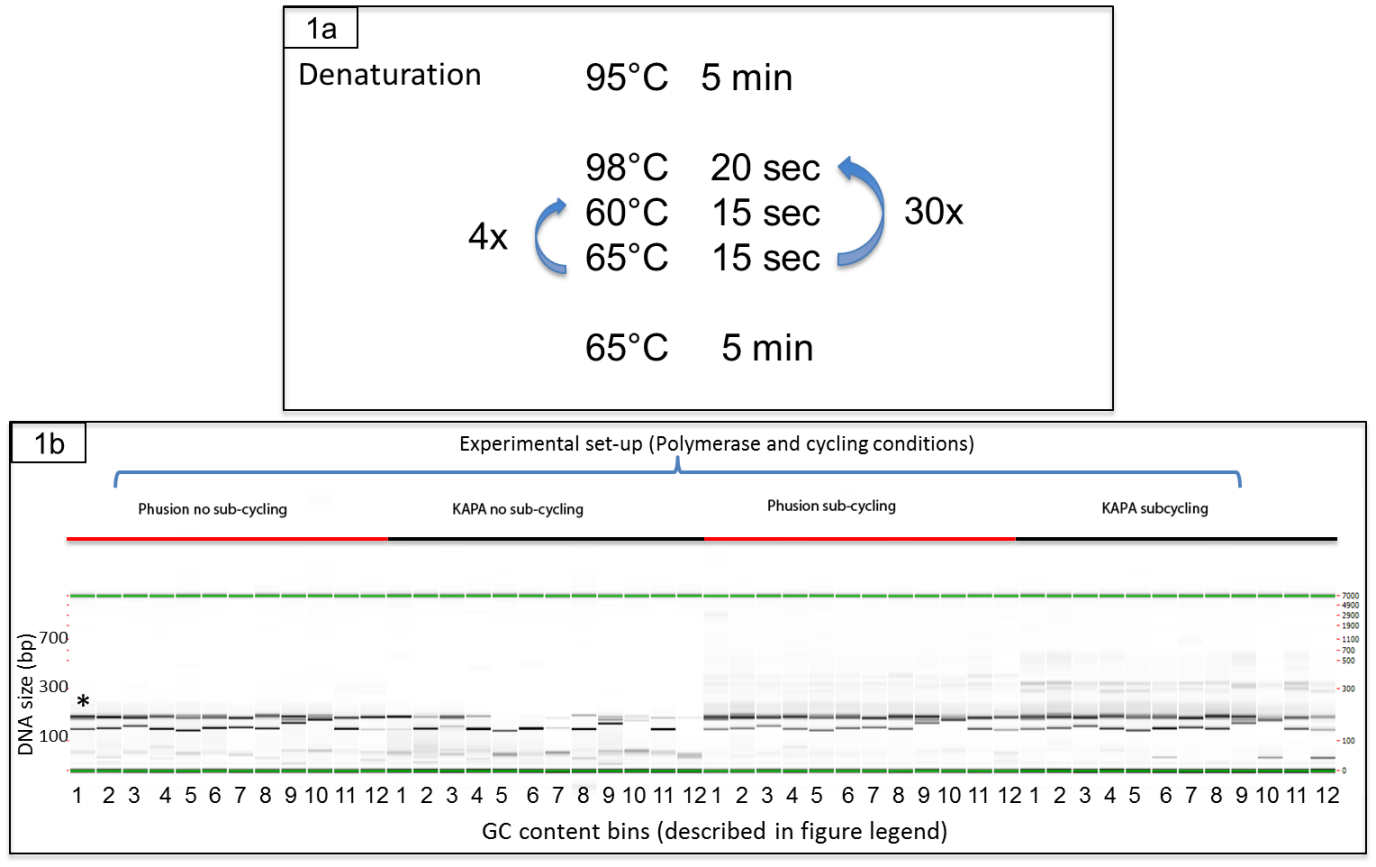
Schematics of the subcycling PCR protocol. A. Each PCR cycle involves a denaturation step and an annealing/elongation step. We introduced 4X sub-cycling the annealing/elongation step within each of the 30X amplification cycles. B. Multiplexed amplification products for pools of 7 oligos with 154–200 bp length products of varying GC content. Phusion and KAPA HIFI polymerases were used with and without a sub-cycling thermocycle. Each different condition is used to amplify 12 separate oligo pools with GC content ranges as follows: 1.) 16.4–34.3; 2.) 13.5–38.7; 3.) 21.5–37.3; 4.) 12.2–12.2; 5.) 12.7–40.0; 6.) 14.9–41.6; 7.) 16.4–37.6; 8.) 12.7–42.0; 9.) 20.9–40.6; 10.) 12.5–42.5; 11.) 12.7–43.7; 12.) 14.8–35.6. Results are based on electronic gels created by electrophoresis using a Perkin Elmer GX instrument with a 5k chip. *Bin shows an example of an expected PCR pattern where a strong 154-200bp product band is seen. PCR reactions were not purified and primers can be seen at the bottom of each sample.

PCR amplification of multiple DNA oligonucleotides containing a wide range of GC content often results in uneven amplification, where a sequence is preferentially amplified over others in a comlplex pool unintentionally, preventing efficient assembly of a complete larger construct. We hypothesized that insertion of a subcycling step during the PCR amplification process would yield a more uniform amplification of all types of oligonucleotides in the mix. To test this hypothesis we introduced a subcycling step that alternates four times between 60°C and 65°C within each one of the regular PCR amplification cycles, as indicated in the scheme in Fig 1a. Using this subcycling protocol we first tested amplification of oligonucleotides consisting of low GC content ranging between 12%-45% GC sequences. Under these conditions we compared amplification using Phusion or KAPA DNA polymerases. KAPA HiFi DNA polymerase was chosen due to its known ability to significantly improve the quality of PCR products from difficult templates containing AT- or GC-rich sequences. As presented in Fig 1b, under normal conditions (i.e. without subcycling), Phusion produced amplified product from all low GC 200bp oligonucleotide templates, while KAPA showed poor amplification for most of the short templates. In contrast, with the subcycling protocol, the amplification of all oligonucleotides was significantly improved for both polymerases, yet there was a clear performance advantage to Phusion (Fig 1b subcycling).

**Fig 2:**
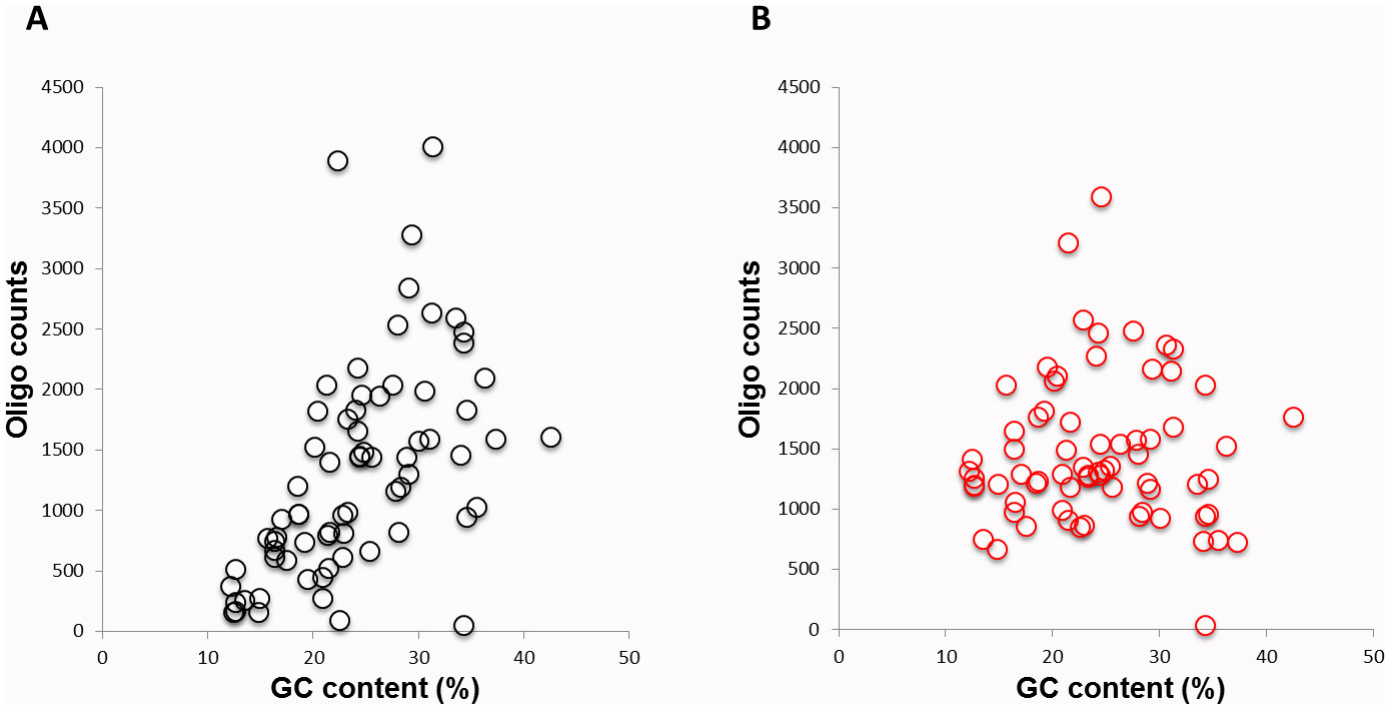
Oligonucleotides copy numbers following multiplex PCR amplification correlated with their GC content. A. Standard PCR amplification; B. Subcycling PCR amplification. *Note*: All oligonucleotides with matching colors and shapes were amplified in the same pool in a multiplexed PCR reaction. Pools were then subjected to a MiSeq analysis and each data point represents the copy number of one oligonucleotide.

To determine whether the subcycling protocol improved the evenness of amplification, pools of 6 oligonucleotides consisting of different GC content (12% to 45% GC) were amplified as a group by multiplex PCR reaction with and without the subcycling protocol. Following the amplification, individual oligonucleotides were subjected to a MiSeq analysis. Fig 2 shows the copy numbers of amplified oligonucleotides from each group. For the data, it is clear that the subcycling protocol greatly improved the amounts of amplified oligonucleotides, and it was specifically efficient for those oligos with GC at the lower end of the spectrum, containing between 12% and 25% GC sequences. Thus, the subcycling protocol resulted in a more even amplification across this range (12%-45% GC).

### Combination of subcycling and 7-deaza-dGTP improves PCR amplification of oligonucleotides with high and low GC content

**Fig 3:**
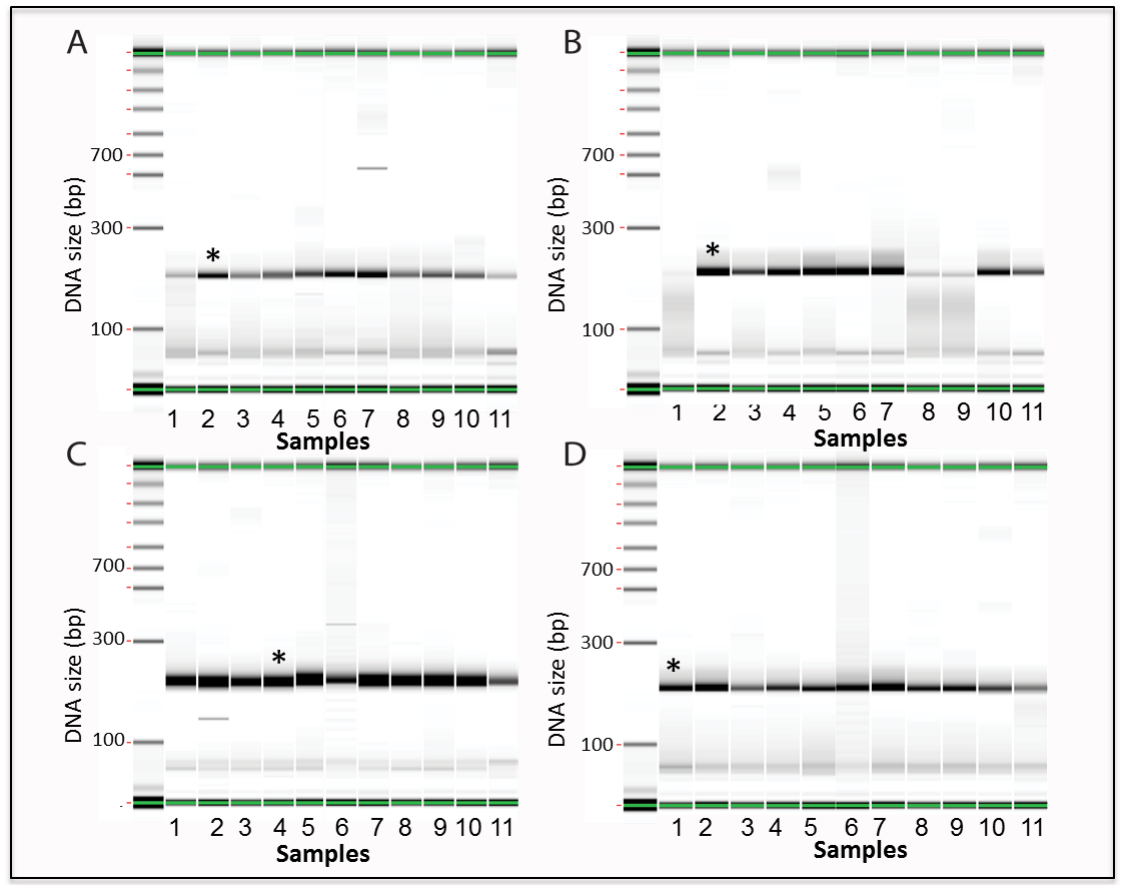
PCR amplification of high and low GC oligonucleotides with and without subcycling and varying additives. A. 10% GC, no sub-cycling. B. 79% GC, no sub-cycling. C. 10% GC, sub-cycling. D. 79% GC, sub-cycling. First lane on the left of each gel: MW markers. Lanes numbered as follows: 1) no additive 2) 40:60 dGTP:deaza-dGTP 3) 50:50 dGTP:deaza-dGTP 4) 60:40 dGTP:deaza-dGTP 5) 2.5% DMSO 6) 10% DMSO 7) 0.1M betaine 8) 0.2M betaine 9) 0.4M betaine 10) 50:50 dGTP:deaza-dGTP with 5% DMSO 11) 60:40 dGTP:deaza-dGTP with 10% DMSO. Results are based on electronic gels created by electrophoresis using a Perkin Elmer GX instrument with a 5k chip. *Bin shows an example of an expected PCR pattern where a strong 200bp product band is seen. PCR reactions were not purified and primers can be seen at the bottom of each sample.

Prior studies report that DMSO and betaine improve PCR amplification of GC-rich DNA when added to the PCR reaction [21,22,23,24]. To examine whether such additives will improve amplification in our study, we used these additives to amplify oligonucleotides with a wider range of GC content (from 10% to 90%), the analysis of this data is shown in Fig 3, and clearly indicate that subcycling improves amplification of both the oligonucleotides containing low GC (10%) and the oligonucleotides with high GC (79%) contents (Fig 3 panels A&B compared to C&D). In addition, combining subcycling PCR with chemical additives, as indicated in Fig 3, yielded complete full-length product and less truncated non-specific products for oligonucleotide templates with low GC range (10% GC) (Fig 3 panels A&C). In contrast, the high GC range (79% GC) templates (Fig 3 panels B&D), showed only slight amplification improvement when subcycling was combined with different additives. Interestingly, the addition of 7-deaza-dGTP (all ranges) and betaine (0.1M) was sufficient to provide strong PCR product without any sub-cycling. Unexpectedly, 0.2M and 0.4M betaine resulted in yield loss when using a standard anneal temperature. Usage of 7-deaza-dGTP did not result in a yield loss, regardless of the final ratio of 7-deaza-dGTP:dGTP used. With sub-cycling, 7-deaza-dGTP and the addition of betaine (Fig 3) reduced the truncated oligonucleotides products and clearly improved the correct amplification of the high GC oligonucleotides (Fig 3 lanes 8&9). In following experiments, the mid-point condition of 0.2M betaine was used in order to maintain balance with sub-cycling while the percentage of 7-deaza-dGTP was maximized (60%). Applying the same experimental conditions in the amplification of oligonucleotides containing GC sequences in the middle range (between 30% and 70% GC), showed only a slight improvement over the normal amplification conditions (data not shown).

**Fig 4:**
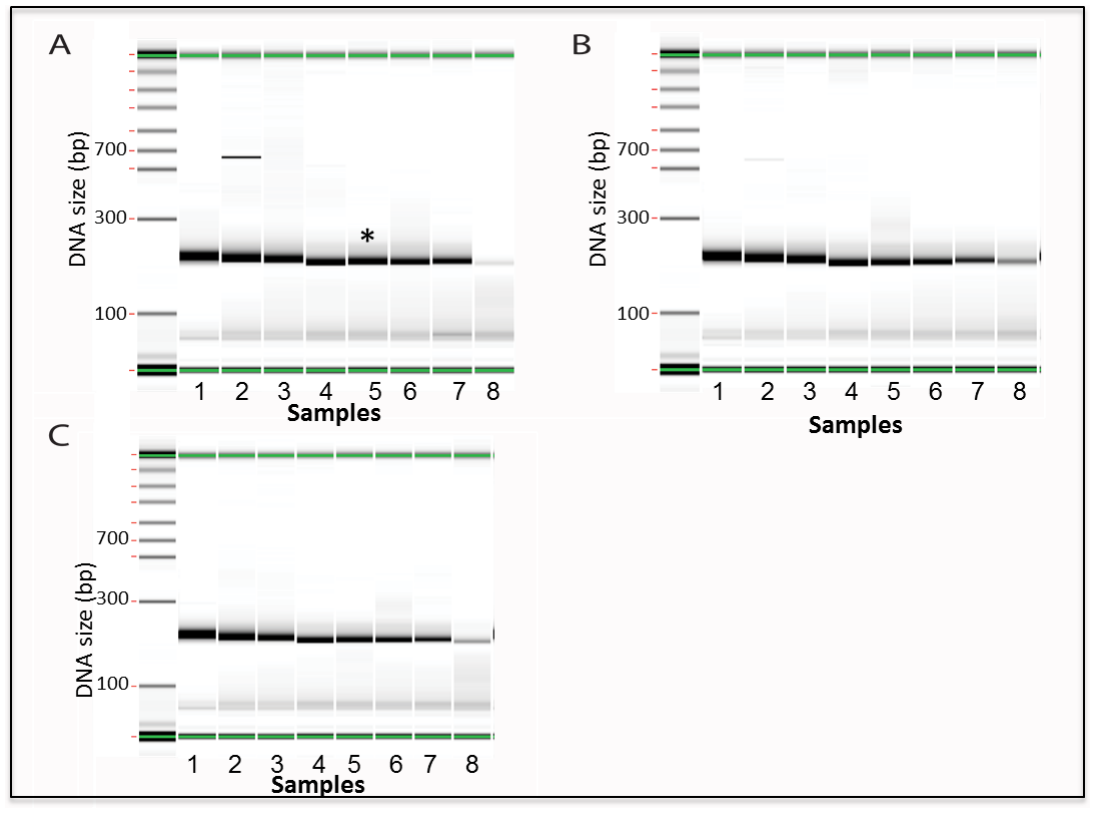
PCR amplification of oligonucleotides with wide range GC contents with subcycling and different additives. A. No additives; B. 60% deaza-dGTP; C. 0.2M betaine. First lane on the left of each gel: MW markers. Lanes numbered indicates oligonucleotides of varying GC content as follows: 1) 10% GC; 2) 21%GC; 3) 33%GC; 4) 44%GC; 5) 56%GC; 6) 67%GC; 7) 79% GC; 8) 90%GC. Results are based on electronic gels created by electrophoresis using a Perkin Elmer GX instrument with a 5k chip. *Bin shows expected PCR pattern where a strong 200bp product band is seen. PCR reactions were not purified and primers can be seen at the bottom of each sample.

In order to define a PCR condition that amplifies the broadest range of GC, we compared amplification of oligonucleotides from 10% to 90% GC using the subcycling protocol combined with the following: no additives, 60% 7-deaza-dGTP, or 0.2M betaine as presented in Fig 4. The results indicate that 60% 7-deaza-dGTP (Fig 4B lane 8 is stronger than that of A and C) when combined with the subcycling protocol, is the best condition, of those listed above, to improve amplification of all tested oligonucleotides and particularly ones with high GC (90%) content.

### Subcycling PCR combined with 7-deaza-dGTP increases the purity and concentration of the synthesized full-length constructs

**Fig 5:**
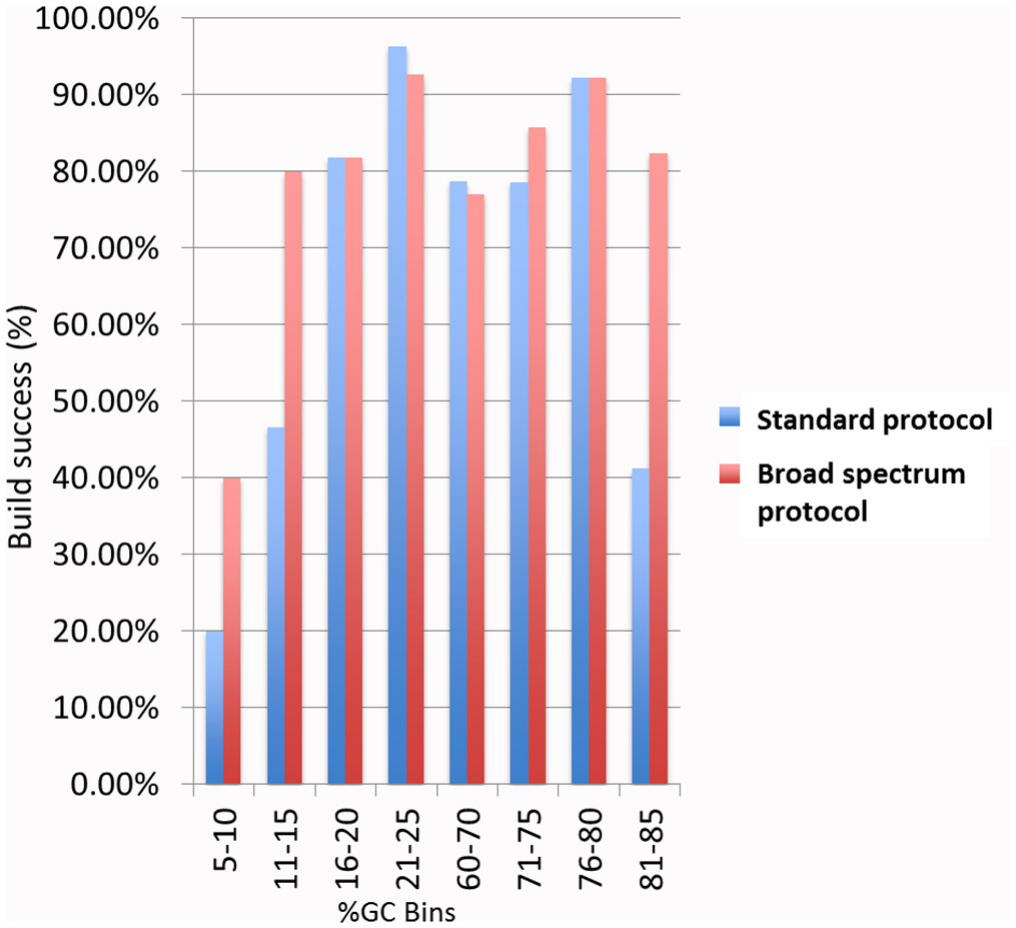
Percent of successful builds of DNA constructs with varying GC content. The bar graph is a visual representation of the data in Tables 1 and 2. Blue bars—represent the successful builds with the standard protocol. Red bars—represent the successful builds with the broad spectrum protocol.

Finally, we sought to establish a quality measurement depicted as build success. To establish that we performed a GX analysis (see methods for details) of the correct full-length constructs following assembly. We compared the correct full—length constructs grouped by their GC content, that were generated by the standard protocol, to the same constructs, generated by the modified, GC protocol. Data are presented as percent of build success by calculating the number of successful assemblies of the correct full-length constructs, per number of attempts, for each of the synthesis protocols. As seen in the Fig 5 bar chart and Table 1, the results clearly indicate that constructs containing 5–10% GC sequences, 11–15% GC, or 81–90% GC sequences, resulted in a decreased build success as compared to sequences with more moderate GC content when the standard protocol was used. However, using the broad spectrum protocol (Fig 5 and Table 2) greatly increased the build success from 20% up to 40% for the constructs containing 5–10% GC, from 46.6% to 80% for the 11–15% GC constructs, and from 41.2% to 82.4% for the constructs containing 81–90%GC. Importantly, the modified GC protocol did not adversely affect the synthesis of constructs containing GC content between 16–80%.

**Table 1:** Percent of successful builds of DNA constructs with varying GC content using the standard protocol. The same data is visualized as a bar graph in Fig 5.

GC Bin	attempts	built	Normal % success
5–10	10	2	20.00%
11–15	15	7	46.67%
16–20	22	18	81.82%
21–25	27	26	96.30%
60–70	61	48	78.69%
71–75	28	22	78.57%
76–80	13	12	92.31%
81–85	17	7	41.18%
totals	193	142	

**Table 2:** Percent of successful builds of DNA constructs with varying GC content using the broad spectrum protocol. The same data is visualized as a bar graph in Fig 5.

GC Bin	attempts	built	GC % success
5–10	10	4	40.00%
11–15	15	12	80.00%
16–20	22	18	81.82%
21–25	27	25	92.59%
60–70	61	47	77.05%
71–75	28	24	85.71%
76–80	13	12	92.31%
81–85	17	14	82.35%
totals	193	156	

## Discussion

The ability to design and synthesize DNA constructs has many applications within molecular biology including the study of large sets of single genes [25], engineering of metabolic pathways for target molecule manufacturing [26], and the ability to safely obtain genes for vaccine research without the need to grow the full pathogens [27,28,29]. All methods of gene synthesis use chemically synthesized small oligonucleotides; however, it is difficult to successfully synthesize DNA sequences that are longer than 150–200 nucleotides by chemical synthesis [19,30,31], One method uses oligonucleotides synthesized on microarray chips. While chip-based synthesis can yield up to10^5^ different sequences per chip, the total synthetic yield of any given oligonucleotide is too low to be used directly in conventional molecular biology methods. Therefore PCR amplification is needed to increase oligonucleotide quantity. However, a major impediment for such PCR amplification is multiple oligonucleotides with variety of GC content including oligonucleotides containing less than 30% GC or higher than 70% GC. Multiplex amplification of such an oligonucleotide mix can be uneven, resulting in low copy numbers of the particular products of the high and low GC oligonucleotides, and becomes a limiting factor for the final assembly of the larger constructs.

Here we show that a combination of subcycling and 7-deaza-dGTP included in the PCR amplification step greatly improve multiplex amplification of oligonucleotides containing a wide range of GC content. During subcycling, the combined annealing/elongation step is composed of subcycles, shuttling between a low and a high temperature, and helps mitigate secondary structure formation. In a similar manner, 7-deaza-dGTP reduces secondary structure formation during PCR as it makes one less chemical contact than dGTP and behaves more like dATP. Our single-shot synthesis process involves multiplex PCR amplification of DNA segments with GC contents from low GC (10%) to high GC (90%). Subcycling PCR was previously reported to improve multiplex PCR amplification of long DNA templates containing regions with low and high GC content [9]. Similarly, we introduced 4X subcycling in the PCR amplification protocol in order to achieve a sufficient amount of amplified synthetic DNA where sequence conservation was critical. We first examined multiple oligonucleotides containing low GC sequences (in the 10%-45% range), and as shown in Fig 1b the subcycling clearly improved the amplification, and produced a significantly higher copy number (Fig 2) of the low GC (12–25% GC) oligonucleotides. In this experiment, we also compared the activity of the two DNA polymerases Phusion and KAPA. Both enzymes offer high fidelity and amplify long templates. In our study, Phusion produced more consistent results for all conditions and oligonucleotides (Fig 1b).

While subcycling improved amplification of oligonucleotide containing low GC (Fig 2), it had less impact on oligonucleotides containing high GC (data not shown). Thus, we combined subcycling with different chemical additives to improve amplification of higher GC material. Recent work by Jensen *et al*. reported that betaine and DMSO when added to the PCR reaction in both the amplification and assembly steps of the synthesis, greatly improved de novo synthesis. In their study, Jensen *et al* use two GC rich templates, the Insulin like growth factor 2 receptor (IGF2R), and the V-raf murine sarcoma viral oncogene homolog B1 (BRAF) [32]. Even though organic chemical additives are known to optimize and improve PCR amplification, very little is known about the precise structural features that make these additives effective [33,34]. We examined the effect of several different additives in multiple concentrations on PCR amplification of DNA oligonucleotides templates containing a wide range of GC sequences (from 10% GC to 90% GC). We found that 7-deaza-dGTP and betaine were both successful in amplifying higher GC material (Fig 3). However, betaine at a high amount (0.2M, 0.4M) resulted in yield loss with a standard anneal temperature for higher GC material. This was an unexpected and repeatable result. We ultimately determined that 7-deaza-dGTP in combination with subcycling significantly improved the correct amplification of all the different oligonucleotides across the board, was less sensitive to cycling conditions and produced more mass than betaine in the PCR for the 90% GC oligonucleotide sequence (Fig 4). As the data presented indicates, most additives do not improve the amplification and can result in truncated shorter products, specifically at the high GC range. To verify the significance of this result we used capillary electrophoresis to compare the correctly assembled long constructs established by the subcycling synthesis protocol, to the ones established by the regular protocol. Capillary electrophoresis allows comparison of size, purity, and concentration of the assembled products. The subcycling protocol greatly improved the synthesis as well as the correct assembly of the long constructs especially for the constructs containing low GC content (5%-15%) and the oligonucleotides containing high GC content (81%-90%), while not significantly affecting the middle range of GC content (16%-80%) constructs (in Fig 5, Tables 1 and 2).

This work provides an updated method for PCR amplification of DNA oligonucleotides containing a broad range of GC sequences without the need of additional protocol modifications or sample extraction and purification. As these challenges are addressed, synthetic biologists will be able to construct next-generation synthetic gene networks with useful applications and advance the game changing areas of regenerative medicine, gene editing and metabolic engineering.

## Supporting Information

S1 File DNA Sequences. MS-Excel spreadsheet containing DNA sequences used in this study.
(XLSX)

## References

[1] Gibson DG, Benders GA, Andrews-Pfannkoch C, Denisova EA, Baden-Tillson H, Zaveri J, et al (2008) Complete chemical synthesis, assembly, and cloning of a Mycoplasma genitalium genome. Science 319: 1215–1220. 10.1126/science.1151721 18218864

[2] Gibson DG, Glass JI, Lartigue C, Noskov VN, Chuang RY, Algire MA, et al (2010) Creation of a bacterial cell controlled by a chemically synthesized genome. Science 329: 52–56. 10.1126/science.1190719 20488990

[3] Tian JD, Ma KS, Saaem I (2009) Advancing high-throughput gene synthesis technology. Molecular Biosystems 5: 714–722. 10.1039/b822268c 19562110

[4] Burbelo PD, Ching KH, Han BL, Klimavicz CM, Ladarola MJ (2010) Synthetic biology for translational research. Am J Transl Res 2: 381–389. 20733948 PMC2923862

[5] Czar MJ, Anderson JC, Bader JS, Peccoud J (2008) Gene synthesis demistified. Trends in Biothechnology 27: 63–72. 10.1016/j.tibtech.2008.10.007 19111926

[6] D'Souza GG, Boddapati SV, Weissig V (2007) Gene therapy of the other genome: the challenges of treating mitochondrial DNA defects. Pharm Res 24: 228–238. 17180727 10.1007/s11095-006-9150-y

[7] Quan J, Saaem I, Tang N, Ma S, Negre N, Gong H, et al (2011) Parallel on-chip gene synthesis and application to optimization of protein expression. Nat Biotech 29: 449–452. 10.1038/nbt.1847 21516083

[8] Sahdev S, Saini S, Tiwari P, Saxena S, Singh Saini K (2007) Amplification of GC-rich genes by following a combination strategy of primer design, enhancers and modified PCR cycle conditions. Mol Cell Probes 21: 303–307. 17490855 10.1016/j.mcp.2007.03.004

[9] Liu Q, Sommer SS (1998) Subcycling-PCR for multiplex long-distance amplification of regions with high and low GC content: application to the inversion hotspot in the factor VIII gene. Biotechniques 25: 1022–1028. 9863056 10.2144/98256rr01

[10] JM M-C, CP B-M, H L-Z, L A-L, MA E-F, B L-G, et al (2007) Frequency of intron 1 and 22 inversions of Factor VIII gene in Mexican patients with severe hemophilia A. Am J Hematol 82: 283–287. 17211847 10.1002/ajh.20865

[11] Ragni M, Ojeifo O, Feng J, Yan J, Hill K, Sommer S, et al (2009) Risk factors for inhibitor formation in haemophilia: a prevalent case-control study. Heamophilia 15: 1074–1082. 10.1111/j.1365-2516.2009.02058.x PMC2734871 19563499

[12] Kilian N, Pospisil V, Hanrahan V (2006) Haemophilia A, factor VIII intron 22 inversion screening using subcycling-PCR. Thromb Heamost 95: 746–747. 16601851

[13] Stirling D (2003) Subcycling PCR for long-distance amplifications of regions with high and low guanine-cystine content: amplification of the intron 22 inversion of the FVIII gene. Methods Mol Biol 226: 101–104. 12958490 10.1385/1-59259-384-4:101

[14] Henke W, Herdel K, Jung K, Schnorr D, Loening SA (1997) Betaine improves the PCR amplification of GC-rich DNA sequences. Nucleic Acids Res 25: 3957–3958. 9380524 10.1093/nar/25.19.3957 PMC146979

[15] Baskaran N, Kandpal RP, Bhargava AK, Glynn MW, Bale A, Weissman S (1996) Uniform amplification of a mixture of deoxyribonucleic acids with varying GC content. Genome Res 6: 633–638. 8796351 10.1101/gr.6.7.633

[16] Frey UH, Bachmann HS, Peters J, Siffert W (2008) PCR-amplification of GC-rich regions: 'slowdown PCR'. Nat Protoc 3: 1312–1317. 10.1038/nprot.2008.112 18714299

[17] Kang J, Lee MS, Gorenstein DG (2005) The enhancement of PCR amplification of a random sequence DNA library by DMSO and betain: application to in vitro combinatorial selection of aptamers. J Biochem Biophys Methods 64: 147–151. 16009429 10.1016/j.jbbm.2005.06.003

[18] Musso M, Bocciardi R, Parodi S, Ravazzolo R, Ceccherini I (2006) Betain, dimethyl sulfoxide, and 7-diaza-dGTP, a powerful mixture for amplification of GC-rich DNA sequences. J Mol Diagn 8: 544–550. 17065422 10.2353/jmoldx.2006.060058 PMC1876170

[19] LeProust EM, Peck BJ, Spirin K, McCuen HB, Moore B, Namsaraev E, et al (2010) Synthesis of high-quality libraries of long (150mer) oligonucleotides by a novel depurination controlled process. Nucleic Acids Res 38: 2522–2540. 10.1093/nar/gkq163 20308161 PMC2860131

[20] Jacobson J, Chu L (2011) Assembly of high fidelity polynucleotides In: USPTO, editor. WIPO. USA: Gen9.

[21] Varadaraj K, Skinner DM (1994) Denaturants or cosolvents improve the specificity of PCR amplification of a G + C-rich DNA using genetically engineered DNA polymerases. Gene 140: 1–5. 8125324 10.1016/0378-1119(94)90723-4

[22] Winship PR (1989) An improved method for directly sequencing PCR amplified material using dimethyl sulphoxide. Nucleic Acids Res 17: 1266 2922271 10.1093/nar/17.3.1266 PMC331767

[23] Frackman S, Kobs G, Simpson D, Storts D (1998) Betain and DMSO: enhanceing agents for PCR. Promega notes: 27–30.

[24] Rees WA, Yager TD, Korte J, Vvon Hippel PH (1993) Betain can eliminate the base pair composition dependence of DNA melting. Biochemistry 32: 137–144. 8418834 10.1021/bi00052a019

[25] Gerhard DS, Wagner L, Feingold EA, Shenmen CM, Grouse LH, Schuler G, et al (2004) The status, quality, and expansion of the NIH full-length cDNA project: the Mammalian Gene Collection (MGC). Genome Res 14: 2121–2127. 15489334 10.1101/gr.2596504 PMC528928

[26] Martin VJ, Pitera DJ, Withers ST, Newman JD, Keasling JD (2003) Engineering a mevalonate pathway in Escherichia coli for production of terpenoids. Nat Biotechnol 21: 796–802. 12778056 10.1038/nbt833

[27] Cello J, Paul AV, Wimmer E (2002) Chemical synthesis of poliovirus cDNA: generation of infectious virus in the absence of natural template. Science 297: 1016–1018. 12114528 10.1126/science.1072266

[28] Smith HO, Hutchison CA 3rd, Pfannkoch C, Venter JC (2003) Generating a synthetic genome by whole genome assembly: phiX174 bacteriophage from synthetic oligonucleotides. Proc Natl Acad Sci U S A 100: 15440–15445. 14657399 10.1073/pnas.2237126100 PMC307586

[29] Hutchison CA, Peterson SN, Gill SR, Cline RT, White O, Fraser CM, et al (1999) Global transposon mutagenesis and a minimal Mycoplasma genome. Science 286: 2165–2169. 10591650 10.1126/science.286.5447.2165

[30] Saaem I, Ma KS, Marchi AN, LaBean TH, Tian JD (2010) In situ Synthesis of DNA Microarray on Functionalized Cyclic Olefin Copolymer Substrate. Acs Applied Materials & Interfaces 2: 491–497. 20356196 10.1021/am900884b

[31] Ma S, Saaem I, Tian J (2012) Error correction in gene synthesis technology. Trends in biotechnology 30: 147–154. 10.1016/j.tibtech.2011.10.002 22209624 PMC3933390

[32] Jensen MA, Fukushima M, Davis RW (2010) DMSO and betaine greatly improve amplification of GC-rich constructs in de novo synthesis. PLoS One 5: e11024 10.1371/journal.pone.0011024 20552011 PMC2883997

[33] Chakrabarti R, Schutt CE (2001) The enhancement of PCR amplification by low molecular-weight sulfones. Gene 274: 293–298. 11675022 10.1016/s0378-1119(01)00621-7

[34] Chakrabarti R, Schutt CE (2001) The enhancement of PCR amplification by low molecular weight amides. Nucleic Acids Res 29: 2377–2381. 11376156 10.1093/nar/29.11.2377 PMC55707

